# The Merits of Dynamic Data Acquisition for Realistic Myocontrol

**DOI:** 10.3389/fbioe.2020.00361

**Published:** 2020-04-30

**Authors:** Andrea Gigli, Arjan Gijsberts, Claudio Castellini

**Affiliations:** ^1^Institute of Robotics and Mechatronics, German Aerospace Center (DLR), Weßling, Germany; ^2^Vandal Laboratory, Istituto Italiano di Tecnologia, Genoa, Italy

**Keywords:** myoelectric control, prosthetic hand, dynamic data acquisition, limb position effect, performance assessment

## Abstract

Natural myocontrol is the intuitive control of a prosthetic limb via the user's voluntary muscular activations. This type of control is usually implemented by means of pattern recognition, which uses a set of training data to create a model that can decipher these muscular activations. A consequence of this approach is that the reliability of a myocontrol system depends on how representative this training data is for all types of signal variability that may be encountered when the amputee puts the prosthesis into real use. Myoelectric signals are indeed known to vary according to the position and orientation of the limb, among other factors, which is why it has become common practice to take this variability into account by acquiring training data in multiple body postures. To shed further light on this problem, we compare two ways of collecting data: while the subjects hold their limb statically in several positions one at a time, which is the traditional way, or while they dynamically move their limb at a constant pace through those same positions. Since our interest is to investigate any differences when controlling an actual prosthetic device, we defined an evaluation protocol that consisted of a series of complex, bimanual daily-living tasks. Fourteen intact participants performed these tasks while wearing prosthetic hands mounted on splints, which were controlled via either a statically or dynamically built myocontrol model. In both cases all subjects managed to complete all tasks and participants without previous experience in myoelectric control manifested a significant learning effect; moreover, there was no significant difference in the task completion times achieved with either model. When evaluated in a simulated scenario with traditional offline performance evaluation, on the other hand, the dynamically-trained system showed significantly better accuracy. Regardless of the setting, the dynamic data acquisition was faster, less tiresome, and better accepted by the users. We conclude that dynamic data acquisition is advantageous and confirm the limited relevance of offline analyses for online myocontrol performance.

## 1. Introduction

*Upper-limb prosthetics*, as a branch of assistive robotics, poses a number of challenges both to robotics and control experts (Vujaklija et al., 2016). A prosthesis is the paradigmatic wearable device since it must be worn during most of the user's daily life. A symbiotic use of such a device, and eventually its embodiment, requires unobtrusive and seamless control (Beckerle et al., 2018a,b; Castellini, 2020). Despite decades of research, such control has not yet been achieved and a widely clinically accepted upper-limb prosthesis has yet to come (Castellini et al., 2014). De facto, the problem consists of several sub-problems—the socket, the sensors, the mechatronics, the appearance, etc.—each one of which must be solved at the same time. Academic solutions, not tested on end-users or at least in realistic conditions, will have little or no impact on the life of disabled users. Upper-limb prosthetics is a deeply holistic problem.

We hereby focus on the myocontrol problem, which is the smooth, multi-DoF control of an upper-limb prosthesis by a user through her voluntary muscle activity. Seamlessly providing the right control commands to a dexterous prosthetic device is an open problem: control based upon biological signals, such as surface electromyography (sEMG) (Merletti et al., 2011), still suffers from clumsiness and unreliability. Although seriously criticized (Schweitzer et al., 2018), the academic solution of choice nowadays is that of collecting labeled biological data from a user engaged in a series of exemplary tasks. This data is then utilized to build a model that maps signals to commands. By the very nature of the approach, it entails that the initial data acquisition phase (of necessarily short duration) must cover the space of all possible muscle configurations that the user will enact in the future (Castellini, 2016). Among other reasons, this is complicated by the so-called *limb position effect* (Fougner et al., 2011; Scheme et al., 2011; Peng et al., 2013), which refers to the change in signals depending on the position and orientation of the limb.

To alleviate this problem, incremental learning and tighter user/prosthesis interaction are generally being studied to improve and complete the initial dataset on demand, while users perform their activities of daily living (ADLs). On the other hand, whenever incremental learning is not used, the limb position effect has been countered by extending the initial data acquisition to include the same action (e.g., a power grasp) carried out in several different postures (Fougner et al., 2011; Geng et al., 2012; Peng et al., 2013; Betthauser et al., 2018). Although this strategy can be effective, it comes at the cost of a considerably longer and more tiresome data acquisition. There have been efforts to limit this increase in acquisition time by replacing a static posture in multiple positions with a single dynamic movement that passes through these positions. For instance, Scheme et al. (2011) have shown that a dynamic protocol not only sped up data acquisition but also improved offline recognition rates during simulated manipulation tasks (e.g., moving an object). An issue with this evaluation is that offline performance is only weakly related to online controllability (Lock et al., 2005; Jiang et al., 2014; Ortiz-Catalan et al., 2015; Hahne et al., 2017; Krasoulis et al., 2019). One of the reasons is that it fails to capture the natural corrections that prosthetic users undertake in response to myocontrol inaccuracies (Hahne et al., 2017).

Recent studies have shown increasing efforts into testing the effects of the data acquisition on realtime myocontrol. Batzianoulis et al. (2018) verified that dynamic training data collected during the reach-to-grasp phase of the prehension improved myocontrol stability during an online pick-and-place task. Similarly, Yang et al. (2017a) and Woodward and Hargrove (2019) acquired training sEMG data while moving the arm and tested the resulting myocontrol models by engaging the participants in online tests derived from, respectively, the target achievement control and the box-and-blocks tests. Both studies confirmed that the performance of myocontrol in online settings improves when the training data is acquired while changing the arm configuration rather than keeping the arm steady in one position. However, none of the studies clarified whether the improved performance was due to recording the dynamic movement of the arm or merely due to the inclusion of more arm poses. The latter study, moreover, also included multiple levels of muscle contractions in the data acquisition, making it impossible to determine the relative contribution of varying the arm position and muscular contraction. More importantly, none of the described performance assessment tests seems to reflect the complexity of everyday actions, since the target achievement control does not involve interactions with real objects, while the box-and-blocks requires performing only one stable grasp in a very limited portion of the user's reachable space. Therefore, it remains unclear if the claimed benefits materialize during complex and realistic ADLs.

*In this work, we characterize the effects of the static and dynamic acquisition of training data on online myocontrol*. In particular, we focus on the loss of controllability associated with variations of the limb position in *realistic daily-living settings*. We asked 14 able-bodied subjects to follow a static and a dynamic data acquisition protocol, while being fitted with two commercially available hand prostheses mounted on splints. With this equipment, and using a myocontrol model built with either statically or dynamically acquired data, they were required to perform a set of bimanual ADLs in a domestic-like laboratory setting. We intentionally employed a bilateral prosthetic setup and chose bimanual tasks to avoid the pitfall of subjects over-relying on their unaffected limb to execute the activities (Chadwell et al., 2018). Furthermore, this also ensures that our study applies equally to a teleoperation scenario.

This work extends the preliminary results we presented at a conference (Gigli et al., 2019) by including the results of a questionnaire, in which the participants evaluated the two data acquisition routines in terms of physical effort and achieved system controllability. Furthermore, we also characterize the learning effect that took place across the participants during the familiarization phase and contextualize the findings of our online experiments with those of previous studies conducted in offline settings. In the following, we thoroughly describe the experimental setup and protocol in 2. The results of our experiment are presented in 3. Further discussion and the conclusions are drawn in 4.

## 2. Materials and Methods

This study emphasizes the importance of a realistic online evaluation of myocontrol. For this reason, we have designed an experiment that involved subjects performing ADLs in a domestic environment, while using a pair of commercially available prosthetic hands. To compare our methodology with that of previous offline studies, we also reused the collected training data for a standard offline grasp recognition experiment. In the remainder of this section, we detail the experimental setup and protocol, the evaluation measures of the online experiment, and the design of the offline analysis.

### 2.1. Participants

Fourteen able-bodied subjects (age 27.9 ± 5.8 years, 10 men and 4 women) were recruited to participate in the experiment. All of them were in good health and none of them had a previous history of disorders that might have influenced the experiment. Four of the participants had prior experience in myocontrol, gained during previous studies, while the others were completely naive to myocontrol. Before the experiment, the subjects received an oral and written description of the experiment and signed an informed consent form. The study was conducted at the German Aerospace Center (DLR) according to the WMA Declaration of Helsinki and approved by DLR's internal committee for personal data protection.

### 2.2. Experimental Setup

**Figure 1:**
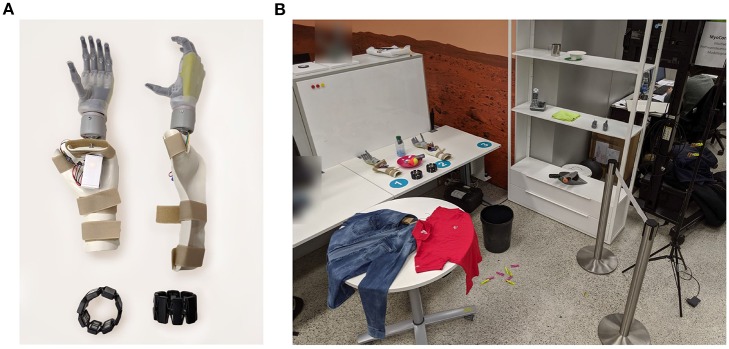
Experimental setup. **(A)** The bimanual prosthetic system consisted of two Myo armbands for sEMG measurement and two Touch Bionics i-Limb Ultra prosthetic hands mounted on orthotic splints. **(B)** The experiment took place in a domestic-like laboratory setting. Tableware, clothes, and other common objects were laid on two tables, three shelves, or on the floor. A clothesline and a vertical support for clothespins were placed next to the shelves.

Each subject wore a *Myo* armband^1^ by Thalmic Labs on both forearms about 5 cm below the elbow. This bracelet contains 8 uniformly spaced sensors, each of which records an sEMG signal at a sampling rate of up to 200 Hz. An orthotic hand/wrist splint was used to hold an *i-LIMB*™ *Revolution* prosthetic hand^2^ by Touch Bionics at the extremity of either forearm. Figure 1A depicts the described hardware. The i-LIMB Revolution comprises six motors under direct independent current control, permitting flexion/extension of each of the five fingers plus abduction/adduction of the thumb. All devices communicated via a serial-port-over-Bluetooth with a laptop that ran the intent detection system. Software on this laptop also guided subjects during data collection, processed the data, trained and ran the controller of each prosthesis. In this manner, an unobtrusive, realistic bimanual prosthetic manipulation setup was implemented, which could be used by unimpaired subjects.

The experiment was conducted in a domestic-like environment, which included some common household objects, two tables, a clothesline, and a system of three shelves. The shelves were placed at a height of 40 cm, 100 cm, and 150 cm. The dining table and the clothesline were placed on the two sides of the shelves. The second table was 2 m away from the clothesline. Certain objects needed some minor modifications to be grasped by the prosthetic hands. The handles of some cutlery, a clothes hanger, and the extremities of small clothespins were padded to grasp them more easily. The study was videotaped for offline performance assessment. An overview of the setup and the environment is shown in Figure 1B.

### 2.3. Data Processing and Training

A custom software suite written in the C# and Python languages was used to acquire, process, and label the input data. The signal from each sEMG channel was rectified, computing its absolute value, and low-pass filtered with a second-order Butterworth filter with a cut-off frequency of 1 Hz. These signals and labels were passed to two instances of non-linear Ridge Regression, one per arm, which were trained with the data of the respective limb. The resulting models mapped sEMG signals onto torque commands for the motors of the prosthetic hands. In its canonical form, Ridge Regression (RR) predicts outputs via a linear model

(1) $$\begin{array}{l} f\left(\text{x}\right)={\text{w}}^{T}\text{x}, \end{array}$$

where **w** is a vector of scalar weights obtained by minimizing

(2) $$\begin{array}{l} \underset{\text{w}}{\min}\overset{N}{\underset{i=1}{\sum }}{\left(y_{i}-f\left({\text{x}}_{i}\right)\right)}^{2}+\lambda {\left\|\text{w}\right\|}^{2} \end{array}$$

over a training set of labeled samples ${\left\{\left({\text{x}}_{i},y_{i}\right)\right\}}_{1}^{N}$. The term ‖**w**‖^2^ penalizes the complexity of the model and its importance relative to minimizing the squared residuals is controlled via the non-negative hyperparameter λ. In the present work, we use a variant of RR that achieves non-linearity by mapping the feature vectors into a high-dimensional representation using so-called Random Fourier Features (RFFs) (Rahimi and Recht, 2008). A detailed treatment of RFF-RR and its use in myocontrol is given in Gijsberts et al. (2014). The prediction function of RFF-RR is written as

(3) $$\begin{array}{l} f\left(\text{x}\right)={\text{w}}^{T}\phi \left(\text{x}\right), \end{array}$$

where ϕ is the finite *D*-dimensional RFF mapping, which consists of cosines weighted through randomly-sampled frequencies. Without going into details, an appealing property of this mapping is that it approximates a Gaussian kernel without incurring the typical computational overhead of actually using that kernel (Rahimi and Recht, 2008), provided that the chosen mapping dimensionality *D* is sufficiently high. The formulation of RFF-RR allows fast training of the model and computation of new predictions, can be made incremental, and is bounded in space (Gijsberts and Metta, 2011), which makes it suitable for realtime myocontrol. Strazzulla et al. (2017), in fact, already used an incremental version of RFF-RR for online bimanual manipulation.

The regularization parameter λ of each regressor was set to 1, while the bandwidth γ and the dimensionality *D* of the RFF mapping to 0.5 and 300, respectively. This regression setup allowed the simultaneous and proportional control of the degrees of freedom (DoFs) of each prosthesis.

### 2.4. Experimental Protocol

The participants donned the prosthetic system, i.e., the sEMG armbands and the prosthetic hands, at the beginning of the experiment, and no doffing or adjustment of the sensors was permitted afterward. This was necessary to isolate the effect of limb position variations from those of other confounding factors, such as the electrode shift.

All subjects in the study tested both the static and dynamic data acquisition protocols. After each data acquisition, the system was trained and the participants were asked to perform a sequence of bimanual activities. This sequence was repeated twice: the first time to let them familiarize themselves with the prosthetic control, the second time to measure their performance. These four segments of the experiment are reported in Table 1. To counterbalance any learning effects, we inverted the order of the acquisition types for half of the subjects: seven randomly selected subjects started with the static acquisition protocol, while the remaining subjects started with the dynamic acquisition protocol.

**Table 1:** Experiment organization.

**Phase#**	**Description**
1	Collect training data using the first acquisition procedure
2	Familiarize on bimanual ADLs
	Measure performance on bimanual ADLs
3	Collect training data using the other acquisition procedure
4	Familiarize on bimanual ADLs
	Measure performance on bimanual ADLs

#### 2.4.1. Data Acquisition

In each data acquisition routine, the participants performed some predefined combinations of grasps and arm postures. After receiving a detailed description of the routines, the participants tried them under the supervision of the experimenter. Then, the experimenter guided them throughout the acquisition procedure, supported by acoustic signals from the acquisition software. This helped to ensure that all subjects performed the same arm configurations and movements. We opted for such direct guidance because the participants did not manage to precisely follow a videotaped execution of the acquisition protocol in preliminary trials. The desired grasp types were chosen based on their relevance in ADLs according to the literature (Wang et al., 2018) and proved to be sufficient to execute our evaluation protocol during preliminary tests. We selected a resting posture, a power grasp, and a pointing posture with the index finger. Since our myocontrol approach was based on proportional control and thus regression, the model was not trained to distinguish these three grasp classes from one another, but rather to predict the corresponding hand configurations in terms of the degree of flexion of each finger. While the participants were performing the grasps during the acquisition phase, the laptop collected the related sEMG samples and labeled them based on whether or not a given DoF was activated in those configurations. In the case of index pointing, the system would record a 0 for the index finger (i.e., no flexion) and 1 for all other DoFs (i.e., flexion). The resting posture consisted of all 0 (all fingers extended), whereas the configuration for the power grasp contained all 1 (all fingers flexed). We intentionally avoided capturing intermediate activation values to avoid the inevitable delay introduced by the subjects' reaction time and to keep the procedure as straightforward as possible for the subjects, which is particularly relevant when considering a possible application with amputees (Sierra González and Castellini, 2013). Moreover, it has been shown that training on binary activation values yields usable proportional control (Gijsberts et al., 2014; Meattini et al., 2019).

We chose a set of limb positions that evenly covered the subject's reachable space, that is, the space they could reach with their hands while standing straight. Since it is uncommon for both hands to be crossed in bimanual activities, we excluded the intersection of the reachable spaces of the left and right hands. To speed up data acquisition, every grasp had to be done with both hands simultaneously, with the arms always symmetric to the sagittal plane. Without loss of generality, we describe the data acquisition routine for one arm only.

#### 2.4.2. Static Protocol

**Figure 2:**
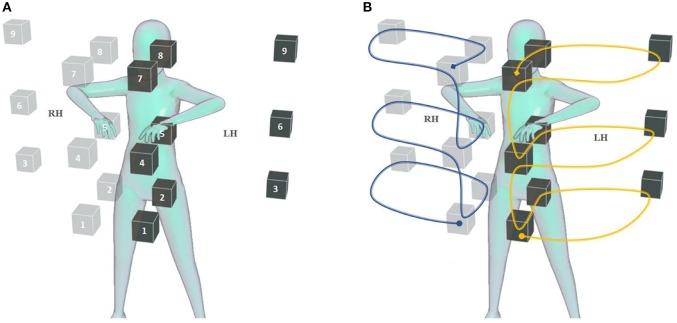
Static and dynamic acquisition of training data. The positions occupied by the right and left hand during data acquisition are represented, respectively, by solid and transparent cubes. **(A)** Static data acquisition was performed repeating and holding the grasp in each position, first with the hand palm facing up, then facing down, for a total of 18 repetitions for each hand. **(B)** During dynamic data acquisition, the grasp was maintained while moving the hands in a trajectory that interpolated the static positions with uniform speed. The trajectory consisted of two halves, from the circle to the square and back; it was followed with the palms down in the first half, and up in the second half. Both data acquisition routines were performed while wearing the bimanual prosthetic system.

During static data acquisition, each grasp was repeated once for a finite set of arm configurations. Previous studies indicated that the robustness of pattern recognition based myocontrol to the limb position effect relates to how well the training data cover the user's workspace in terms of reachable positions (Fougner et al., 2011; Radmand et al., 2014) and possible forearm rotations (Khushaba et al., 2016; Yang et al., 2017b). For this work, we selected 18 arm configurations that seemed to represent a good trade-off between a homogeneous sampling of the workspace and the duration of the resulting data acquisition. They corresponded to reaching nine positions with the hand, first with the palm facing down and then with the palm facing up (see Figure 2A). We defined these positions based on three height levels (waist, chest, head) and three relative distances from the trunk (close in front, far in front, far lateral). We believe that this definition favors the repeatability of the arm configuration across different subjects since it relates to one's own body rather than to external references. Each grasp was held in every position for 3 s, which was the lowest duration found in similar studies (Fougner et al., 2011; Radmand et al., 2014; Khushaba et al., 2016), and 4 s were allowed to move the arm from one configuration to the next. The acquisition of each grasp type took 126 s in total, 54 s to record data, and 72 s to reach the different arm configurations. In the case of fatigue, the participants were allowed to pause the routine and rest.

#### 2.4.3. Dynamic Protocol

In the dynamic data acquisition, the subject performed the desired grasp type with both hands while moving the arms in a trajectory that interpolated the nine positions of static acquisition, as shown in Figure 2B. The movement started from the waist level with the palm down and proceeded upward, passing through all nine positions. Then the subject flipped the hand palm up and continued the movement backward until she returned to the starting position. This movement was repeated once for each grasp type, while the corresponding data was recorded. Its duration was chosen to be 27 s, exactly half the recording time of the static acquisition, and 4 s were allotted to prepare the following grasp. Even in this case, the participants could suspend the procedure to rest.

#### 2.4.4. Activities of Daily Living

After processing the data and training the prosthesis controllers, we evaluated the system by having the subjects perform the bimanual ADLs that are described in Table 2. These activities were inspired by those found in assessment protocols for prosthetic users, such as ACMC (Hermansson et al., 2005) and SHAP (Kyberd et al., 2009), and for patients with motor impairments of the upper limbs, like CAHAI (Schuster et al., 2010) and the Clothespin relocation test (Hussaini and Kyberd, 2017). We preferred tasks that involved coordinated movements of the arms or walking and bending, as these were more susceptible to the limb position effect. The experimenter explained the tasks to the participants before the familiarization phase. Unless otherwise specified, participants could autonomously choose which grasp to use to carry out a certain task. For example, they could open the bottle cap by grabbing it or pushing its edge with the tip of their index finger. No constraint was imposed on task laterality, that is, which hand was to be used to perform a particular action. During pick and place tasks, the subjects could decide to move one or two objects at the same time depending on the amount of trust they had in the prostheses. The tasks proceeded without time limits and it was the subjects' responsibility to recover from errors, such as an accidental drop of an object. An exception was made for the last task where the experimenter replaced the clothespins anytime they were dropped on the floor.

**Table 2:** Detailed description of the bimanual tasks in the performance evaluation phase.

**Task**	**Name**	**Description**
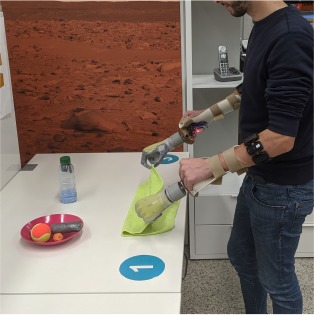	Napkin	A napkin is placed on the middle shelf, unfolded. Take it, bring it to the dining table, and fold it twice.
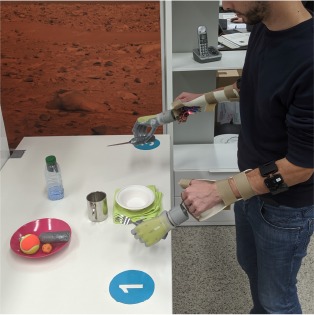	Table	A plate and a glass are laid out next to each other on the top shelf. Bring them to the dining table, put the plate on the folded napkin and the glass next to it. A fork and a knife are on the middle shelf. Bring them to the dining table and place them on the two sides of the plate. Move two objects at the same time if the prostheses seem reliable.
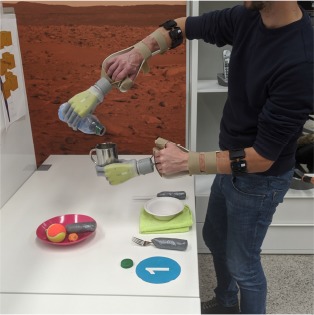	Water	A bottle containing some fine gravel is on the dinner table. Pick it up with one hand, unscrew the cap with the other hand, pour the gravel into the glass, put the bottle back on the table with the cap next to it.
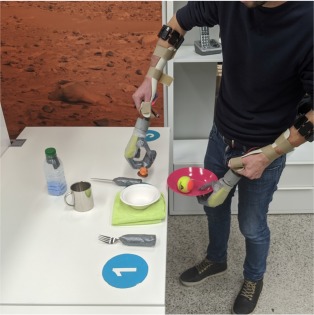	Food	A spoon and two small balls with diameters of 3 and 6.5 cm are contained in a bowl that is placed on the dining table. Take the plate with one hand and the spoon with the other, then use the spoon to bring the balls from the bowl to the plate.
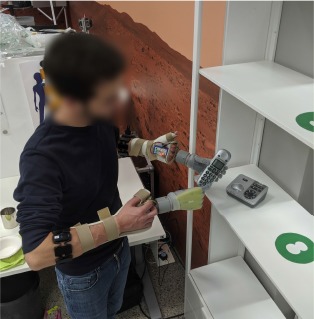	Phone	A cordless phone is connected to its base station on the middle shelf. Take it with one hand, dial 9-1-1 with the index finger of the other hand, and then put the phone back in place.
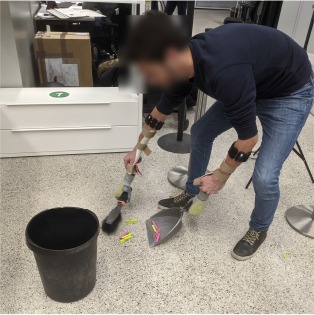	Sweep	A hand broom and a dustpan are positioned on the lower shelf, while some clothespins lie on the floor next to a trash bin. Take the broom with one hand and the dustpan with the other, walk to the clothespins, bend, and sweep the clothespins off the floor. Then empty the dustpan into the trash bin and bring the broom and dustpan back to their original location.
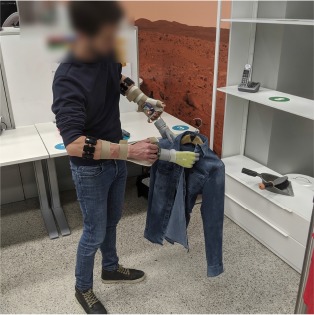	Shirt	A dress shirt and a hanger are placed on the table. Use both hands to put the shirt on the hanger, then hang the hanger on the clothesline.
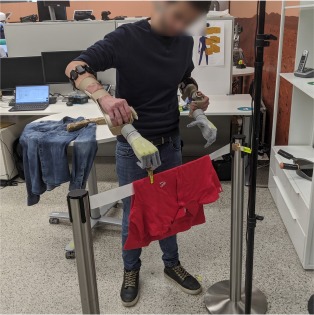	T-shirt	A t-shirt is positioned on a table and two clothespins are pinned to a vertical rod in front of the clothesline. Pick the t-shirt up with two hands, bring it to the clothesline, put it on the wire, and pin it with the clothespins.

### 2.5. Online Performance Evaluation

The effectiveness of the two data acquisition routines was evaluated quantitatively by measuring the completion time of the individual tasks during the performance test phase. Since we did not impose any time limits, the completion rate of the tasks was by definition 100%. At the end of the experiment, the participants were requested to fill in a questionnaire with two questions to qualitatively investigate potential differences between both acquisition types. The subjects were first asked to report how easy they found it to control the system on a visual analog scale ranging from “very difficult” to “very easy.” Secondly, they had to quantify how comfortable it was to complete either data acquisition, on a similar visual analog scale from “very tiring” to “very comfortable.” The effort made during data acquisition was also quantified indirectly by measuring the amount of time a subject requested to rest during data acquisition.

We expected to find differences in the task completion times and the perceived levels of fatigue associated with the two data acquisition routines. We used a two-tailed Wilcoxon signed-rank test to identify statistically significant differences between the average task completion times and the perceived fatigue of the two procedures. The choice of this test was based on the limited number of participants and the within-subject study design. The significance threshold was set to α = 0.05.

### 2.6. Offline Grasp Prediction

To compare our methodology with related literature, we also conducted an offline analysis that reflects the study by Scheme et al. (2011). For every combination of subject, arm, and acquisition method, we partitioned the data of the acquisition phase in training and test sets. In the static case, we assigned the data of the odd-numbered of the 18 arm positions to the training set and the even-numbered ones to the test set. For the static case, which consisted of a continuous motion rather than a set of discrete positions, we approximated the same split by first dividing the entire data sequence into 18 parts of equal length. This particular split was chosen to minimize leakage from the test set to the training set due to temporal dependencies, while at the same time limiting the distribution shift between both sets.

Distinct RFF-RR models were trained for all four datasets (static and dynamic, left and right arm) of thirteen subjects, where we note that one subject was excluded from the offline analysis because the data had not been stored correctly. These models were trained in the same manner and with the same hyperparameters as for the online experiment. Their performance was then evaluated on the test set of the same type (e.g., static to static) as well as across acquisition types (e.g., static to dynamic). How well a model performed was quantified by averaging the unadjusted coefficient of determination *R*^2^ obtained for the predicted activation levels of each DoF. The coefficient *R*^2^ for one DoF is defined as

(4) $$\begin{array}{l} R^{2}\left(\text{y},\hat{\text{y}}\right)=1-\frac{\sum_{i=1}^{N}{\left(y_{i}-{ŷ}_{i}\right)}^{2}}{\sum_{i=1}^{N}{\left(y_{i}-ȳ\right)}^{2}} \end{array}$$

where $\hat{\text{y}}$ is a vector of *N* predictions, **y** is the corresponding ground truth, and ȳ is the average value of the ground truth.

## 3. Experimental Results

We compared the two data acquisition procedures based on the physical effort of the participants, the time needed to complete the manipulation tasks using the resulting myocontrol models, and the perceived controllability of the prosthetic system. We then evaluated the effects of our methodology in offline settings to compare it with previous works in the field.

**Figure 3:**
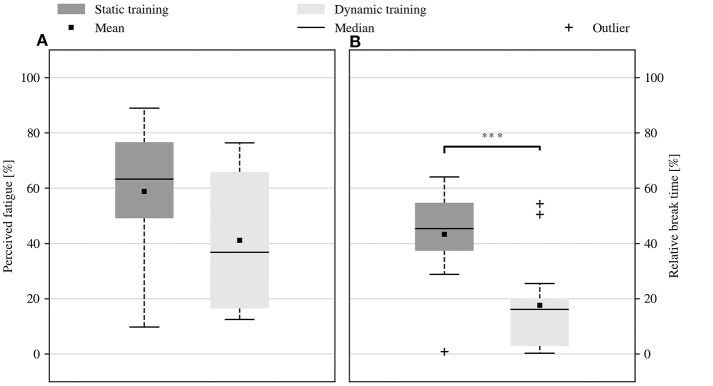
Physical effort required by static and dynamic data acquisition. **(A)** Self-reported level of fatigue experienced during data acquisition. **(B)** Proportion of the data acquisition duration that was spent resting. The dynamic acquisition proved to be less tiring since it required significantly less break time (^***^p < 0.001). The outcome of the questionnaire seemed to confirm this result, but it was not supported by sufficient statistical evidence. The reduction of break time also allowed to collect dynamic data much faster than static data. In all the boxplots of this study, the rectangle indicates the range between the first and third quartiles, and the whiskers extend to the most extreme samples within the first and the third quartile ∓ 1.5 IQR. Samples outside this range are marked as outliers.

Figure 3 quantifies the physical effort needed to complete the data acquisition. The perceived level of fatigue was derived from how comfortable static and dynamic acquisition were evaluated in the questionnaire, by converting the answers into a percentage from 0% (“very comfortable”) to 100% (“very tiring”). Additionally, since the subjects could suspend the data acquisition in case of weariness, a complementary metric of fatigue was obtained by measuring the proportion of acquisition time spent while resting. The subjects showed no agreement on which strategy required the least physical effort. Although the reported fatigue was lower for dynamic training, this result was not statistically significant (average level of fatigue of 58.9% vs. 41.1%, *p* = 0.078, *V* = 81). It must be noted, however, that dynamic training required significantly shorter break times (43.3% vs. 17.6% of the overall data acquisition duration on average, *p* < 0.001, *V* = 105). Taken together, these results indicate that dynamic training was indeed less tiring. Furthermore, they suggest that the discomfort during static acquisition was compensated by taking longer breaks, which would also justify the mixed opinions found in the questionnaires. Remarkably, the shorter break times made dynamic acquisition significantly faster than static acquisition, especially considering that it was already shorter by design.

**Figure 4:**
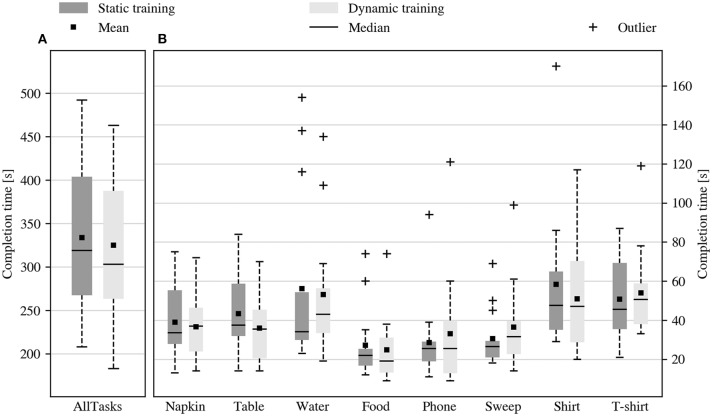
Duration of the tasks during the performance evaluation session. Duration of **(A)** the task sequence and **(B)** of the individual tasks during the performance evaluation session, after static and dynamic training. No significant difference was found between the average duration of the performance evaluation session in the two conditions.

The real-time performance of the prosthetic system was assessed based on the time it took subjects to complete the tasks in the performance evaluation session that followed the data acquisition. Figure 4 reports the performance of all the subjects after static and dynamic training. The duration of the evaluation session was comparable after either acquisition procedure (mean task sequence duration of 333.8 s vs. 325.1 s, *p* = 0.855, *V* = 49). Particularly, also the completion times of the individual tasks were comparable (no statistically significant difference), regardless of their different requirements in terms of dexterity and movement coordination.

**Figure 5:**
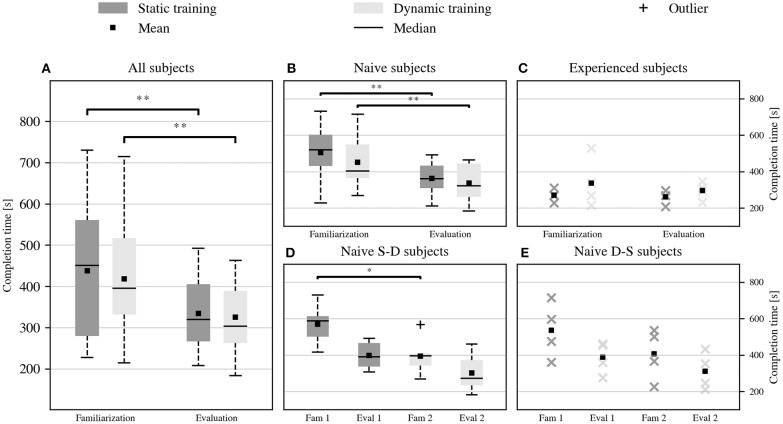
Duration of the tasks sequence during the familiarization and the performance evaluation sessions, after static and dynamic data acquisition. **(A)** Performance of all the participants. Although the task completion time decreased significantly after the initial familiarization (^**^p < 0.01), the performance of static and dynamic data acquisition remained comparable both during the familiarization and the following evaluation session. This trend characterized the performance of **(B)** naive participants but not those of **(C)** experienced participants, for whom the average session duration did not change significantly across acquisition strategies and familiarization levels. The performance of the naive participants rapidly converged to those of the experienced ones, despite retaining a higher variance. **(D,E)** Chronological evolution of the performance of the naive subjects, divided between those who first tested the static and then the dynamic condition, and vice versa. The improvement of all the naive subjects was consistent, not only within the same training strategy but also across different strategies (^*^p < 0.05). For groups with fewer than five samples, we show the individual data points rather than a boxplot.

Figure 5A reports the average duration of the familiarization and the performance evaluation sessions that followed either data acquisition. The order for the static and dynamic training was randomized among subjects to counterbalance possible learning effects between the two strategies. The subjects demonstrated a strong learning effect, as they completed the evaluation session significantly faster than the familiarization session, both after static (average session duration of 438 s vs. 334 s, *p* = 0.0012, *V* = 100) and dynamic training (average session duration of 418 s vs. 325 s, *p* = 0.007, *V* = 94). The evaluation session also showed reduced variability in duration across the subjects compared to the familiarization session, both in the static (session duration range of 503 s vs. 284 s) and in the dynamic case (session duration range of 501 s vs. 280 s). Nonetheless, the two data collection procedures showed comparable task completion times during the respective familiarization and evaluation sessions (no statistically significant difference). In other terms, the subjects' performance improved rapidly over time due to practice, but this improvement occurred independently of the data acquisition procedure.

The analysis of the learning effect continued by separating the performance of naive and experienced subjects, and then by dividing the naive subjects based on who tested the static acquisition followed by the dynamic acquisition, defined as naive SD subjects, or vice versa, defined as naive DS subjects. Three of the four experienced subjects belonged to the SD group. Of the remaining ten naive subjects, six were SD and four DS. Naive participants, Figure 5B, confirmed the learning trend described before, showing comparable performance across training conditions while improving over time (average duration of the familiarization and the evaluation session after static training 505 s vs. 363 s, *p* = 0.002, *V* = 45, and after dynamic training 451 s vs. 337 s, *p* = 0.0098, *V* = 52). Experienced participants, instead, performed equivalently well regardless of the training condition and did not show a significant learning effect (average duration of the familiarization and the evaluation session after static training 269 s vs. 260 s, and after dynamic training 337 s vs. 296 s). The performance of naive subjects was characterized by a higher initial variance, but it seemed to converge rapidly to that of experienced participants over the course of the experiment. Figures 5D,E display the evolution over time of the performance of naive SD and naive DS participants. In both groups, the familiarization of the second tested condition was faster than that of the first condition (average familiarization time for naive SD subjects 570 s vs. 394 s, *p* = 0.031, *V* = 21; average familiarization time for naive DS subjects 537 s vs. 407 s, *p* = 0.12, *V* = 10). The lack of statistical evidence in the second case was probably due to the limited number of DS subjects. This result showed that learning did not just happen within the same training condition, but rather that the subjects transferred some of the skills acquired for the first training strategy to the second. This transfer effect could explain part of the variability of the counterbalanced results, especially during the familiarization phase.

**Figure 6:**
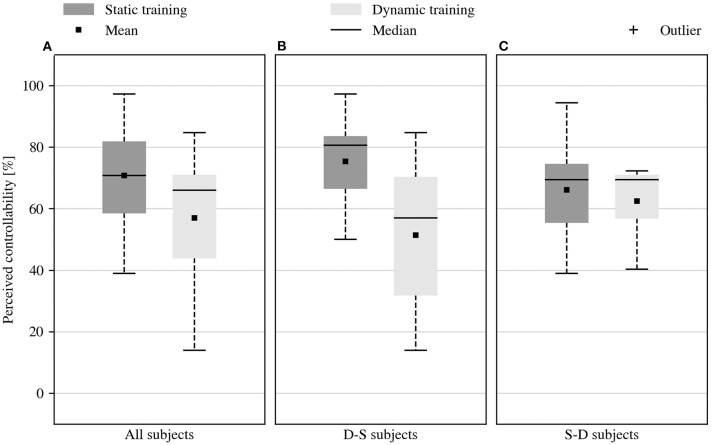
Perceived controllability of the system. **(A)** Controllability of the system, self-assessed in the questionnaire at the end of the experiment. Subjects reported mixed opinions about the controllability of the prosthetic system. No statistical evidence supported the modest improvement of the perceived controllability provided by the static training. **(B)** The subjects who started the experiment with dynamic training and continued with static training *(D-S)* reported improved system controllability for the second condition tested. **(C)** Those who tried the static training first *(S-D)* experienced equivalent controllability in both conditions.

Figure 6A describes how easy the subjects perceived the two prosthetic control variants during the online tasks. This information was reported in the questionnaire at the end of the experiment and converted in a percentage from 0% (“very difficult”) to 100% (“very easy”). The subjects' opinions were mixed, which overall resulted in a comparable perceived system controllability after either acquisition strategy. Nonetheless, the perceived controllability of the system was higher after static training, but this was not supported by the statistical evidence (average controllability of 70.8% vs. 57.0%, *p* = 0.059, *V* = 72.5). Furthermore, this trend seemed to characterize only a portion of the subjects. Those who tried the static training after the dynamic one, Figure 6B, consistently reported improvements in the usability of the system for the last tested condition (average controllability of 75.4% vs. 51.4%, *p* = 0.063, *V* = 20). Instead, the subjects that started with static training, Figure 6C, found that controlling the system was equally easy under both conditions (average controllability of 66.1% vs. 62.5%, *p* = 0.61, *V* = 17.5). In any case, none of the observed effects was statistically significant, perhaps because the opinions regarding the first tested condition were always characterized by greater variance.

**Figure 7:**
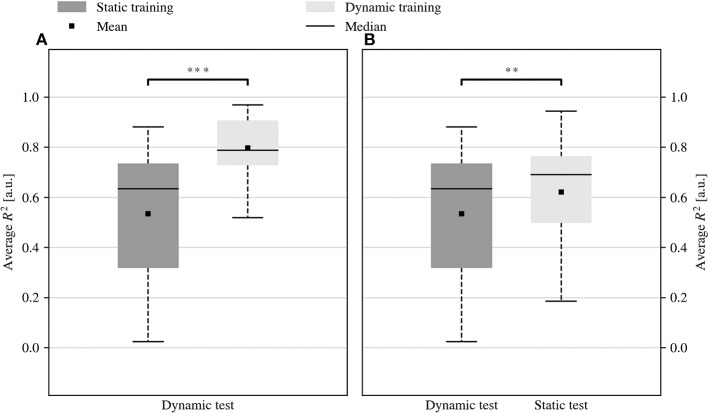
Offline prediction of the hand configuration using RFF-RR after static or dynamic training. **(A)** Dynamic training allowed better grasp prediction from dynamically acquired data samples (^***^p < 0.001). Besides, **(B)** the performance observed by training on dynamic and testing on static data were better than those obtained by training on static and testing on dynamic data (^**^p < 0.01).

Figure 7 summarizes the outcomes of the offline grasp recognition task performed on the training data collected during the online experiments. The prediction of desired hand configurations in the dynamic test set was significantly better after dynamic training as compared to static training (*R*^2^ of 0.53 vs. 0.80, *p* < 0.001, *z* = −4.46, see Figure 7A). In addition, the dynamic training provided better performance also when the training and the test data were acquired with different protocols, i.e., static training followed by dynamic testing, or dynamic training followed by static testing. Figure 7B shows that the estimation of the intended hand posture obtained by training on dynamic data and testing on static data was better than the estimation obtained by training on static and testing on dynamic data (*R*^2^ of 0.53 vs. 0.62, *p* = 0.004, *z* = −2.86).

## 4. Discussion and Conclusions

### 4.1. General Remarks

The limb position effect requires training data to be collected in several different body postures for each desired action to be learned; this is because body postures alter the muscle configuration of the forearm, thereby changing the sEMG patterns. The traditional solution to this problem, already appearing multiple times in literature (Fougner et al., 2011; Peng et al., 2013; Betthauser et al., 2018), consists of simply asking users to hold their arm statically in multiple postures and then collecting data one posture at a time. This makes the data collection procedure longer and potentially more tiring than usual, especially since this procedure must be repeated for each grasp type. A method to make it lighter and faster is highly desirable.

The aim of this work was that of *assessing if a dynamic data-collection procedure would be better than a static one* and, if so, in which respect and why. We wanted to *test the models obtained using either data collection procedure in realistic conditions*, i.e., using prosthetic hardware to perform real-time bimanual manipulation tasks inspired by daily living. Fourteen able-bodied subjects were engaged in a set of realistic bimanual activities (laying a table, serving food, hanging clothes, etc.), after having performed both a static and a dynamic data collection procedure to build appropriate myocontrol models.

The first result to be noted is that all users were able to complete all tasks, in both training modalities. Given the realism of the tasks they were requested to perform, this seems to indicate that the approach of using RR with RFFs is worth pursuing. Notice that in this specific work we intentionally refrained from using the incremental characteristics of RFF-RR, making it impossible to update and correct the models online; the observed performance, therefore, only depends on the data collected at the beginning of the experiment. Secondly, it is worth remarking that the time needed to acquire the training data is relatively short in either modality. Taking into account the breaks requested by the subjects, the average acquisition time is about 9 min for the static acquisition and just 2 min for the dynamic one. Also allowing for the adaptation that users naturally put in place while doing the tasks, this indicates that both approaches rapidly yield data with a quality sufficient to cover most of the actions required in the experiment.

### 4.2. Dynamic and Static Data Collection Provide Comparable Real-Time Performance

The experimental results show that, quite surprisingly, there is no difference in real-time performance (time required to complete each task) between the static and the dynamic data collection procedure. No statistically significant difference in the task execution times could be found, either considering the overall times, or the duration of the individual tasks (Figure 4).

Notice as well (Figure 5) that subjects without prior experience in myocontrol manifest a quite strong learning effect as they perform the tasks over and over again. However, the uniformity between the static and dynamic conditions persists, since there is no significant difference in performance between the familiarization phases, as well as between the evaluation phases. The results for the experienced participants suggest that long-term learning of myocontrol leads subjects to reach a consistent level of performance that is irrespective of the acquisition protocol. This reduction in variability among experienced subjects is in line with the findings of previous studies on the implications of long-term user training on myocontrol (Hargrove et al., 2017).

The equivalence between the myocontrol performance provided by static and dynamic training somehow contradicts the outcome of previous studies (Fougner et al., 2011; Scheme et al., 2011), which reported improved myocontrol in offline settings by using dynamic training data. To the best of our knowledge, however, this is the first time in which the comparison between the effects of static and dynamic training is carried out online, performing realistic and complex ADLs. In line with the results by Scheme et al. (2011), we observe that in the cross-comparison in Figure 7B the model trained on dynamic data has higher offline accuracy on static data than vice versa. All in all, this result suggests once more that non- or quasi-realistic testing of myocontrol systems is hardly a good indicator for the efficacy or reliability of the system once put to practice in real life (Jiang et al., 2014; Ortiz-Catalan et al., 2015). This might be due to many contingent reasons, such as wrong measures of performance or wrong tasks administered to the users, but eventually it probably has to do most of all with the excellent ability of human users to compensate for control inaccuracies by adapting their muscular signals. This is even more so for proportional control since users receive immediate visual feedback of the control response of the prosthesis (Hahne et al., 2017; Shehata et al., 2017, 2018).

Considering the capability of users to smoothen control inaccuracies, one may wonder if this also means that we can shorten the data acquisition even further, for instance by reducing the number of positions. A recent study on real-time myocontrol did not find a reduction in the online grasp recognition rate in different positions even when training data was acquired in just one position (Hwang et al., 2017). This study did not involve realistic tasks and considered only one wrist orientation and three positions; regardless, in future work, it would be interesting to continue along these lines and to investigate what is the minimal amount of position variability that still yields consistent online controllability during practical tasks.

### 4.3. Dynamic Data Collection Is Faster and Less Tiresome

As is clear from the objective and self-assessed indices of performance, acquiring data dynamically is faster, uses fewer sEMG data, and is less tiring. Net of the possible break times requested by the participants, the dynamic procedure only takes 27 s per grasp type instead of the 126 s needed by the static one; this is advantageous in terms of the stress and frustration imposed on the subjects. Moreover, from a computational point of view, dynamic training employs roughly half the sEMG samples needed by the static one. This could be important when miniaturization of the whole system is planned, for instance on a microcontroller to be embedded in the prosthetic socket. Interestingly, while providing fewer data samples, dynamic acquisition still results in equivalent real-time controllability to the static one. We argue that this depends on the information captured during the motion that joins one limb posture to the following one, which is ignored by performing static acquisition in multiple postures.

The subjective assessment of fatigue during either procedure represents one of the main results of this study. Although not statistically significant, the results in Figure 3A hint that the dynamic acquisition was perceived as less tiring. This observation is supported by the amount of rest the subjects requested during either type of acquisition, shown in Figure 3B, which was significantly less for the dynamic procedure. This indicates that dynamic training is easier and more acceptable than the static one. Taken together, the two results indicate that dynamic training should be preferred over static training.

### 4.4. Further Remarks

According to the visual inspection of the recordings of the experiments, and also according to the main experimenter's experience, the myocontrol system was not free from instabilities and failures. For example, the prosthetic hands would sometimes execute unwanted actions or open unexpectedly during grasps. Mainly, these problems arose when trying to grasp while in muscle-stressing body postures, probably akin but not exactly matching those during data collection. Since we did not allow subjects the possibility to update the models online, this indicates that there still is some incompleteness of the dataset collected at the beginning of the experiments. In other words, it cannot be assumed that an initial calibration will suffice (Castellini, 2016).

The solution we propose to address this issue is, once again, the exploitation of the incremental characteristics of RFF-RR (Strazzulla et al., 2017), leading to interactive learning (Nowak et al., 2018). Notice that there is no conflict in mixing up interactive learning as described in the literature and dynamic data collection. These two strategies are orthogonal and one can imagine updating the model online already during the dynamic data acquisition. This would provide the user with immediate feedback on the control response of the prosthesis; going even one step further, the user could then guide the acquisition and interactively acquire data exactly in those postural and dynamical conditions where the behavior is unsatisfactory.

A complementary avenue to attenuate the limb position effect consists of enriching the training set with sensory modalities that directly relate to the position of the arm. Fougner et al. (2011) showed that offline myocontrol accuracy can be improved by integrating sEMG and accelerometry data collected in multiple arm positions. Radmand et al. (2014) later found that the use of inertial measurements in combination with static data acquisition only improves myoelectric control if the training data is acquired across many arm positions, whilst it is likely to undermine the grasp recognition performance if a suboptimal set of training positions is selected. When the training data is acquired dynamically, instead, inertial measurements prove beneficial for myocontrol quality even if the user's workspace is not thoroughly sampled. Finally, more recent studies confirmed that the dynamic acquisition of myographic and inertial training data improves the myocontrol performance also in online settings (Krasoulis et al., 2017).

This experiment was conducted with able-bodied subjects only, although we put them in conditions that closely mimic the everyday life of prosthetic users. How much do our results apply to subjects with an amputation? Although the answer can only be found by testing our methodology on amputated users, it seems reasonable to argue that our main result, that dynamic acquisition is quicker and more comfortable than a static one, can directly transfer to amputees—less muscular stress is always good, as long as it does not hinder performance. The range of muscle movement after an amputation is generally limited, and the distribution of the weight of the limb across the muscular structure can be dramatically different between amputees and intact users; this is a further hurdle toward the translation of our results to amputees. Nevertheless, both acquisition strategies presented in this paper could as well be tailored to each individual, also for transhumeral or even lower-limb amputees. In principle, the advantage of dynamic over static training should hold also when a tailored training protocol is designed. Lastly, sensor-shift during limb motion can be problematic for amputees and may have been mitigated in our setup. In fact, while biosignal sensors are normally integrated into the prosthetic socket and may be slightly affected by its movement, we used a tight sEMG armband that is independent of the prosthetic splint. In our experience, however, sensor-shift can be reduced effectively with a well-designed, bespoke socket that would still make the two strategies equivalent.

Last but not least, the approach shown in this work can, and probably should, be applied in realms other than upper-limb prosthetics; for instance, to control rehabilitation devices for patients of musculoskeletal degenerative conditions. Stroke survivors, for instance, might benefit from a faster data collection procedure, when engaged in rehabilitation procedures involving complex robotic devices. Rehabilitation based upon Virtual Reality is also a target to this procedure (Nissler et al., 2019). Robotic control based upon muscle activity can be also transferred to teleoperated scenarios (Porges et al., 2019) and, probably, in space. In all these scenarios it is worth investigating the usefulness and feasibility of the procedure described in this paper.

### 4.5. Conclusions

To summarize, to try and solve the limb position effect in myocontrol we have investigated an alternative to the classic multi-body-posture data collection. Namely, we have compared it with a dynamic data acquisition procedure, which consists in collecting data while the user was moving the arm smoothly through all the postures. To test the true controllability resulting from either procedure, we have designed a realistic evaluation protocol that required the subjects to perform a set of bimanual activities of daily living. Our results show that the two procedures yield similar performance, but that dynamic training is faster and less tiresome. This seems to indicate that the dynamic acquisition procedure should be preferred over the static one.

## Data Availability Statement

The datasets generated for this study are available on request to the corresponding author.

## Ethics Statement

The studies involving human participants were reviewed and approved by DLR's internal committee for personal data protection and conducted according to the WMA Declaration of Helsinki. The patients/participants provided their written informed consent to participate in this study.

## Author Contributions

AGig had the original idea for the study, designed and carried out the user study under the supervision of CC, and performed the analysis under the supervision of AGij and CC. All authors contributed to the design of the study, discussed the results, and wrote the manuscript.

## Conflict of Interest

The authors declare that the research was conducted in the absence of any commercial or financial relationships that could be construed as a potential conflict of interest.

## Footnotes

^1^https://support.getmyo.com/hc/en-us/articles/203398347-Getting-started-with-your-Myo-armband

^2^https://www.ossur.com/en-us/prosthetics/arms/i-limb-ultra
